# Margin status after breast-conserving surgery for breast cancer in the Netherlands from 2009 to 2022

**DOI:** 10.1093/bjsopen/zrag063

**Published:** 2026-06-09

**Authors:** Joni J Nijveldt, Bas Keizers, Sabine Siesling, Anne Brecht Francken, Kiran K Rajan, Alexander H de Haas, Bert van der Vegt, Linetta B Koppert, Schelto Kruijff, Pieter J van der Zaag, Wendy Kelder, NBCA Consortium, Martinus A Beek, Henriette A Schuttevaer, Thomas Schok, Hinne A Rakhorst, Liesbeth Jansen, Marjan van Hezewijk, Carolien H M van Deurzen, José H Volders, Ilse Jannink, Margrethe S Schlooz-Vries, Ernst J P Schoenmaeckers, Marije C Gordinou de Gouberville, Alwine A Hellingman, Emily L Postma, Ester J M Siemerink, Floris P R Verbeek, Marian B E Menke-Pluijmers, Joost Nonner, Vivianne C G Tjan-Heijnen, Yvonne L J Vissers, Linda de Munck, Djaëlla V Hooiveld, Carlijn T I de Betue, Dominique J P van Uden, Marianne R F Bosscher, Marieke E Straver, Carla Meeuwis, Caroline M E Contant, Enja J Bantema-Joppe, Milou H Martens, Miriam L Hoven-Gondrie, Léonie H M Smit, Marthe Brinckman, Gea A Gooiker

**Affiliations:** Department of Surgery, University Medical Centre Groningen, Groningen, the Netherlands; Department of Surgery, Isala, Zwolle, the Netherlands; Department of Surgery, University Medical Centre Groningen, Groningen, the Netherlands; Department of Nuclear Medicine and Molecular Imaging, University Medical Centre Groningen, Groningen, the Netherlands; Department of Health Technology and Services Research, Technical Medical Centre, University of Twente, Enschede, the Netherlands; Department of Research and Development, Netherlands Comprehensive Cancer Organization (IKNL), Utrecht, the Netherlands; Department of Surgery, Isala, Zwolle, the Netherlands; Department of Surgery, Isala, Zwolle, the Netherlands; Department of Radiology, Isala, Zwolle, the Netherlands; Department of Pathology, Academic Breast Centre Groningen, Martini Hospital, Groningen, the Netherlands; Department of Pathology and Medical Biology, Academic Breast Centre Groningen, University Medical Centre Groningen, Groningen, the Netherlands; Department of Surgery, Erasmus MC Cancer Institute, Rotterdam, the Netherlands; Department of Surgery, University Medical Centre Groningen, Groningen, the Netherlands; Department of Nuclear Medicine and Molecular Imaging, University Medical Centre Groningen, Groningen, the Netherlands; Department of Molecular Medicine and Surgery, Karolinska Institutet, Stockholm, Sweden; Department of Nuclear Medicine and Molecular Imaging, University Medical Centre Groningen, Groningen, the Netherlands; Molecular Biophysics, Zernike Institute, University of Groningen, Groningen, the Netherlands; Department of Surgery, Academic Breast Centre Groningen, Martini Hospital, Groningen, the Netherlands

## Abstract

**Background:**

Tumour-positive margins after breast-conserving surgery (BCS) for breast cancer increase the risk of local recurrence and require additional therapy. Intraoperative imaging techniques in high-risk patients could improve margin determination. This study describes margin status after BCS in the Netherlands between 2009 and 2022, focusing on trends in surgical margin status and identifying subgroups at higher risk of tumour-positive margins.

**Methods:**

All patients undergoing BCS for non-metastatic breast cancer in the Netherlands from 2009 to 2022 were selected from the Netherlands Cancer Registry. Data included patient and tumour characteristics, treatment details, and surgical margin status. Descriptive statistics and trend analyses were performed. Univariate and multivariable analyses were performed to identify risk factors and patient subgroups at higher risk of margin involvement. Marginal effects analyses quantified tumour-positive margin risks.

**Results:**

In total, 109 475 women were included in the study. The mean tumour-positive margin rate was 10.8%. An extensively positive margin (> 4 mm involvement) occurred in 3.9% of patients, with a decreasing trend seen until 2013. Risk factors for a positive margin were invasive lobular carcinoma, multifocal disease, clinical tumour (cT) category 2 or 3, and neoadjuvant chemo- or hormonal systemic therapy without pathological complete response (pCR), with odds ratios (ORs) ranging from 1.56 to 2.96. One or more of these risk factors was present in 44 772 patients (40.9% of total cohort). The probability of positive margins increased with the number of risk factors, from 6.8% (no risk factors) to 49.1% (all four risk factors). Preoperative understaging (cT<pathological tumour) was also associated with an increased margin risk (OR 3.69). Two prediction tools were developed based on these outcomes (for total positive margins and for extensively positive margins only).

**Conclusion:**

Over the past decade, tumour-positive margin rates in the Netherlands have remained stable at 10.8%. Patients with invasive lobular carcinoma, cT2/3 tumours, multifocal disease, or those receiving neoadjuvant chemotherapy or hormonal systemic therapy without pCR remain at substantially higher risk. There is room for improvement in these patient subgroups, highlighting the need for intraoperative imaging innovations to reduce positive margins.

## Teaser

Tumour-positive margins after breast-conserving surgery increase the risk of local recurrence and require additional therapy. In the Netherlands, between 2009 and 2022, positive margin rates remained stable at 10.8%, with substantially higher risks in patients with invasive lobular carcinoma, clinical tumour category 2/3 tumours, multifocal disease, or patients receiving neoadjuvant chemotherapy or hormonal therapy without pathological complete response. There is room for improvement in these patient subgroups.

## Introduction

Breast cancer treatment requires a multidisciplinary approach. Despite improved systemic and radiotherapeutic management, surgical removal of tumour tissue remains the cornerstone of curative treatment. Increasingly, personalized surgical therapy is provided, based on tumour characteristics and patient preferences. For T1 to T2 (≤ 5 cm) tumours, the preferred approach is breast-conserving surgery (BCS), in which only the tumour is resected along with a small rim of healthy tissue to obtain clear margins, followed by radiation therapy. For tumours unsuitable for BCS, mastectomy used to be standard treatment. However, due to advances in neoadjuvant systemic therapies^1,2^ and oncoplastic techniques^3^, BCS is more often feasible nowadays.

Although BCS benefits patients due to smaller wounds, fewer wound infections, and better cosmetic outcomes than mastectomy, less extensive surgery increases the risk of incomplete resection^4–6^. Aside from a psychological burden, tumour-positive margins increase the risk of local recurrence and reduce overall survival, necessitating additional therapy^7,8^. Since 2002, the Netherlands has followed a national guideline for invasive breast cancer that deviates from international practices^9^. Whereas the international consensus of negative margins remains as ‘no ink on tumour’, the Netherlands national guideline distinguishes between ‘focally positive’ (≤ 4-mm tumour margin involvement) and ‘extensively positive’ (> 4-mm tumour margin involvement or multiple focally positive foci) surgical margins. Although patient preferences and shared decision-making may alter additional treatment choice, re-excision is recommended for extensively positive margins, whereas patients with focally positive margins can opt for a radiation therapy boost or re-excision^9^. This focally positive margin approach has been shown to result in acceptable and similar disease-free and overall survival rates compared with initial complete resection^10^. Despite reducing the disadvantages of positive margins, additional treatment increases morbidity and reduces quality of life. Consequently, achieving complete resections during initial surgery is vital.

Today, surgeons use preoperative imaging, often combined with markers or tumour localization guidewires placed before surgery^5^. Sometimes, perioperative imaging with ultrasound is added^11^. Although these techniques are essential for guiding the procedure, they fail to provide information regarding resection margins. As a result, intraoperative determination of margins is challenging even for experienced surgeons, because it primarily relies on tactile and visual information available to the surgeon. To improve margin determination, intraoperative imaging techniques offering real-time feedback, such as fluorescence imaging, are being explored^12–14^. However, to understand the potential impact of these innovations, examining current trends in margin status after BCS is essential. Therefore, the aim of this study was to determine trends in margin status after BCS for non-metastatic breast cancer in the Netherlands from 2009 to 2022. Furthermore, the aim was to identify specific patient subgroups at increased risk of tumour-positive margins after BCS.

## Methods

### Patient selection

Women treated with BCS for non-metastatic breast cancer in the Netherlands from 2009 to 2022 were selected from the Netherlands Cancer Registry. This nationwide registry is hosted by the Netherlands Comprehensive Cancer Organization (IKNL) and registers all newly diagnosed cancer patients in every hospital in the Netherlands, based on a national pathology databank (PALGA^15^) and national discharge register notifications. Data is collected by well-trained data managers from medical records and made available for research upon request. Exclusion criteria included pathological complete response (pCR) after neoadjuvant systemic therapy, ductal carcinoma *in situ* (DCIS) only, pathological T4 (pT4) tumours, preoperative radiotherapy, surgery other than BCS, missing year of surgery, and incomplete margin status.

### Clinicopathological data

Data comprised patient and tumour characteristics, treatment information, and surgical margin status. Patient characteristics included age and year of diagnosis. Tumour characteristics consisted of tumour node metastasis (TNM) staging system classification, localization, histological tumour type, modified Bloom and Richardson differentiation grade^16^, hormone receptor (HR) status (negative oestrogen (ER)/progesterone (PR) receptor status defined as < 10% expression), human epidermal growth factor receptor 2 (HER2) status (divided into groups according to immunohistochemistry (IHC): IHC 0/1+, HER2negative; IHC 2+, equivocal HER2 expression; and IHC 3+, HER2 positive), multifocality, the presence of DCIS component, and palpability. Receptor subgroups were defined by HR/HER2 status. Data regarding fluorescence *in situ* hybridization or silver *in situ* hybridization in patients with equivocal IHC for HER2 status were not available. Therefore, patients with an equivocal HER2 receptor status were included in the category ‘unknown’ and were excluded from subsequent analyses. Treatment information contained preoperative magnetic resonance imaging (MRI) scan (yes/no) and type of neoadjuvant systemic treatment (none, chemotherapy, hormonal therapy, and/or targeted therapy). Margin status was based on pathology reports, distinguishing negative, focally positive (≤ 4-mm tumour margin involvement), and extensively positive (> 4-mm tumour margin involvement or multiple focally positive foci) margins.

### Statistical analysis

Descriptive statistics were used to provide insights into patient and tumour characteristics. The significance of differences in clinicopathological and treatment characteristics were evaluated using χ^2^ tests. Trends for margin status over the years are shown graphically, distinguishing focally positive and extensively positive margins. To identify patient subgroups at higher risk of tumour-positive margins, univariate analysis calculated log odds ratios (ORs) for each individual variable known before surgery. Subsequently, multivariable analyses were performed to adjust for confounders. First, binary logistic regression analysis was conducted, in which both focally positive and extensively positive margins were seen as tumour-positive margins. Second, multinomial logistic regression analysis was performed with focally positive and extensively positive margins as separate categories. Stepwise regression was used to assess changes in significance.

The binary logistic regression analysis and multinomial logistic regression analysis were used to develop web-based prediction tools for tumour-positive margins, based on β coefficients. Calibration of the models was assessed using the intercept. Subsequently, logistic regression was used to assess the association between specific risk factors identified in multivariable analyses and the occurrence of positive surgical margins. Based on high-risk factors identified by multivariable analyses, the adjusted probabilities of tumour-positive margins were determined using a composite risk count. These probabilities were estimated using marginal effects, and 95% confidence intervals were calculated by bootstrap resampling with 1000 replicates. All outcomes are presented with their 95% confidence interval (c.i.), and *P* < 0.05 was considered statistically significant. Missing values were categorized as unknown. Observations with unknown data were excluded from analyses.

Graphs were generated using GraphPad Prism version 9.0.0 (GraphPad Software Inc, San Diego, California, USA). Statistical analyses were conducted using Stata^®^ Release 17 (StataCorp, College Station, Texas, USA).

## Results

**Fig. 1:**
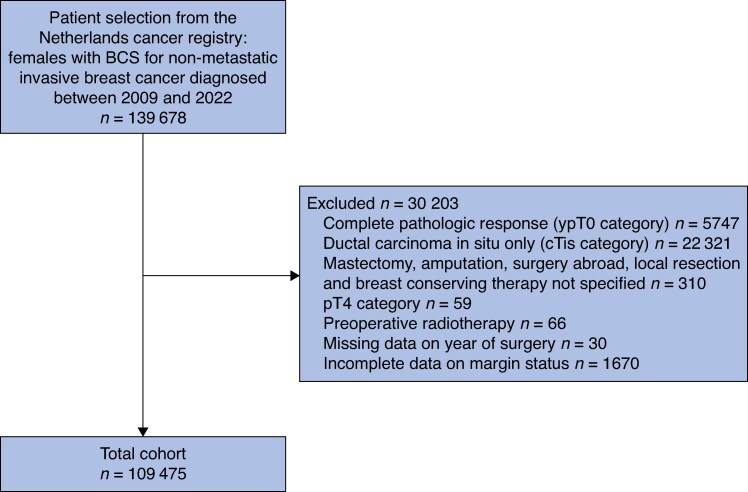
Flow chart of patient inclusion in the study BCS, breast-conserving surgery; yp, post-neoadjuvant therapy; cTis, clinical tumour *in situ*; pT, pathological tumour.

The database contained data for 139 678 women; of these, 30 203 were excluded, resulting in a cohort of 109 475 patients for analysis in the present study (*Fig. 1*).

### Patient and tumour characteristics

Patient and tumour characteristics, in total and according to margin status, are presented in *Table 1*. The median age of patients at the time of BCS was 62 (interquartile range 53–70) years, and most patients had clinical tumour (cT) category 1 tumours (71.4%). The most common histological tumour type was invasive carcinoma of no special type, formerly known as invasive ductal carcinoma (82.7%). Unifocal carcinoma was present in 90.9% of patients, 47.1% of patients had tumours with differentiation grade 2, and 88 166 patients (80.5%) had HR+/HER2− tumours.

**Table 1:** Clinicopathological and treatment characteristics overall and according to margin status of women treated with breast-conserving surgery for non-metastatic breast cancer in the Netherlands from 2009 to 2022

	Total cohort	Negative margins	Focally positive margins†	Extensively positive margins‡	P*
Total no. patients	109 475 (100.0%)	97 658 (89.2%)	7585 (6.9%)	4232 (3.9%)	
**Age (years)**					< 0.001
< 40	2806 (2.6%)	2470 (88.0%)	215 (7.7%)	121 (4.3%)	
40–49	13 208 (12.1%)	11 461 (86.8%)	1064 (8.1%)	683 (5.2%)	
50–75	83 390 (76.2%)	74 849 (89.8%)	5540 (6.6%)	3001 (3.6%)	
> 75	10 071 (9.2%)	8878 (88.2%)	766 (7.6%)	427 (4.2%)	
**Histological type**					< 0.001
No special type	90 578 (82.7%)	82 321 (90.9%)	5539 (6.1%)	2718 (3.0%)	
Invasive lobular carcinoma	11 090 (10.1%)	8529 (76.9%)	1467 (13.2%)	1094 (9.9%)	
Mixed type	2899 (2.7%)	2299 (79.3%)	336 (11.6%)	264 (9.1%)	
Other	4908 (4.5%)	4509 (91.9%)	243 (5.0%)	156 (3.2%)	
**Multifocal disease**					< 0.001
No	99 514 (90.9%)	89 697 (90.1%)	6393 (6.4%)	3424 (3.4%)	
Yes	9612 (8.8%)	7676 (79.9%)	1165 (12.1%)	771 (8.0%)	
Unknown	349 (0.3%)	285 (81.7%)	27 (7.7%)	37 (10.6%)	
**cT category**					< 0.001
T1	81 158 (74.1%)	74 035 (91.2%)	4777 (5.9%)	2346 (2.9%)	
T2	26 885 (24.6%)	22 702 (84.4%)	2576 (9.6%)	1607 (6.0%)	
T3	1432 (1.3%)	921 (64.3%)	232 (16.2%)	279 (19.5%)	
**pT category**					< 0.001
T1	84 745 (77.4%)	78 025 (92.1%)	4716 (5.6%)	2004 (2.4%)	
T2	22 691 (20.7%)	18 260 (80.5%)	2657 (11.7%)	1774 (7.8%)	
T3	835 (0.8%)	253 (30.3%)	175 (21.0%)	407 (48.7%)	
Unknown	1204 (1.1%)	1120 (93.0%)	37 (3.1%)	47 (3.9%)	
**Differentiation grade**					< 0.001
1	30 625 (28.0%)	28 270 (92.3%)	1601 (5.2%)	754 (2.5%)	
2	51 561 (47.1%)	45 023 (87.3%)	4157 (8.1%)	2381 (4.6%)	
3	22 912 (20.9%)	20 667 (90.2%)	1439 (6.3%)	806 (3.5%)	
Unknown	4377 (4.0%)	3698 (84.5%)	388 (8.9%)	291 (6.7%)	
**DCIS component**					< 0.001
No	48 718 (44.5%)	43 376 (89.0%)	3448 (7.1%)	1894 (3.9%)	
Yes	45 522 (41.6%)	41 017 (90.1%)	3036 (6.7%)	1496 (3.2%)	
Unknown	15 235 (13.9%)	13 265 (87.1%)	1101 (7.2%)	869 (5.7%)	
**Tumour palpable**					< 0.001
No	31 245 (28.5%)	28 763 (92.1%)	1688 (5.4%)	794 (2.5%)	
Yes	39 027 (35.7%)	34 159 (87.5%)	3135 (8.0%)	1733 (4.4%)	
Unknown§	39 203 (35.8%)	34 736 (88.6%)	2762 (7.1%)	1705 (4.4%)	
**Localization**					< 0.001
Lateral	54 147 (49.5%)	48 567 (89.7%)	3642 (6.7%)	1938 (3.6%)	
Medial	23 811 (21.8%)	21 412 (89.9%)	1578 (6.6%)	821 (3.5%)	
Central	6547 (6.0%)	5696 (87.0%)	522 (8.0%)	329 (5.0%)	
Other	24 970 (22.8%)	21 983 (88.0%)	1843 (7.4%)	1144 (4.6%)	
**Receptor status**					< 0.001
HR+/HER2−	88 166 (80.5%)	78 115 (88.6%)	6 444 (7.3%)	3 607 (4.1%)	
HR+/HER2+	7487 (6.8%)	6702 (89.5%)	519 (6.9%)	266 (3.6%)	
HR−/HER2+	2335 (2.1%)	2162 (92.6%)	119 (5.1%)	54 (2.3%)	
HR−/HER2−	9523 (8.7%)	8906 (93.5%)	391 (4.1%)	226 (2.4%)	
Unknown	1964 (1.8%)	1773 (90.3%)	112 (5.7%)	79 (4.0%)	
**Preoperative MRI scan**					< 0.001
No	73 264 (66.9%)	66 342 (90.6%)	4530 (6.2%)	2392 (3.3%)	
Yes	36 211 (33.1%)	31 316 (86.5%)	3055 (8.4%)	1840 (5.1%)	
**Neoadjuvant systemic therapy#**					< 0.001
No	95 715 (87.4%)	86 521 (90.4%)	6057 (6.3%)	3137 (3.3%)	
Chemotherapy	8250 (7.5%)	6625 (80.3%)	943 (11.4%)	682 (8.3%)	
Hormonal therapy	2841 (2.6%)	2160 (76.0%)	373 (13.1%)	308 (10.8%)	
Targeted therapy	2669 (2.4%)	2352 (88.1%)	212 (7.9%)	105 (3.9%)	

Values in parentheses are percentages. †Defined as ≤ 4-mm tumour margin involvement. ‡Defined as > 4-mm tumour margin involvement or multiple focally positive foci. §Registered until 2019. #Patients with complete response after neoadjuvant systemic therapy (ypT0) were excluded (most of these patients had ER/PR/HER2-negative tumours: ER–, 62.5%; PR–, 75.5%; and HER2–, 53.6%). cT, clinical tumour; pT, pathological tumour; DCIS, ductal carcinoma *in situ*; HR, hormone receptor; HER2, human epidermal growth factor receptor 2; MRI, magnetic resonance imaging; ER, oestrogen receptor; PR, progesterone receptor. *χ^2^ test.

### Margin status after BCS

Margins were tumour-positive in 11 817 patients (10.8%; *Table 1*); 7 585 patients (6.9%) had a focally positive margin and 4 232 patients (3.9%) had an extensively positive margin. The highest positive margin rates, focally and extensively positive margins combined, were observed in patients with invasive lobular carcinomas (23.1% invasive lobular carcinoma only; 18.7% mixed-type), cT2/3 stage tumours (cT2, 15.6%; cT3, 35.7%), pT2/3 stage tumours (pT2, 19.5%; pT3, 69.7%), multifocal disease (20.1%), and after neoadjuvant systemic chemotherapy (19.7%) or hormonal therapy (24.0%) without pCR. Of patients with tumour-positive margins after neoadjuvant systemic therapy, 92.4% were ER positive.

### Margin status over time

**Fig. 2:**
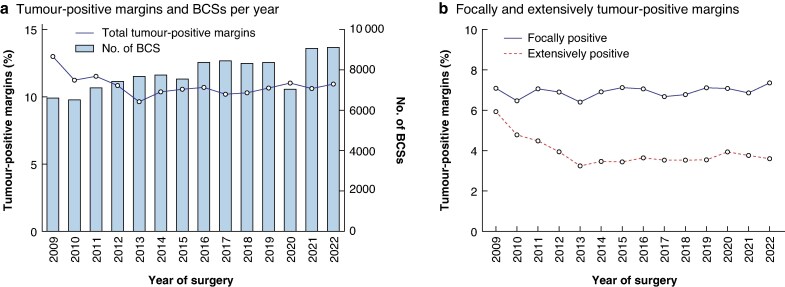
Tumour-positive margin rates in women treated with BCS for non-metastatic breast cancer in the Netherlands from 2009 to 2022 **a** Overall tumour-positive margin rates over the time and the number of BCSs performed per year. **b** Overall tumour-positive margin rates per year for focally positive and extensively positive margins. BCS, breast-conserving surgery.

The number of BCSs performed increased from 6600 in 2009 to almost 9200 in 2022. Yet, the tumour-positive margin rate decreased from 13.0% in 2009 to 11.0% in 2022 (*Fig. 2a*). This declining trend was mainly observed in 2009–2013, after which tumour-positive margin rates stabilized at 10.6%. When analysed in more detail, mean(standard deviation) focally positive margin rates remained constant at 6.9(0.3)% (*Fig. 2b*), with extensively positive margins appearing to contribute primarily to the overall decline in tumour-positive margin rates, which decreased from 5.9% in 2009 to 3.3% in 2013 and remained stable at 3.6(0.2)% thereafter.

### High-risk subgroups for tumour-positive margins

Univariate analysis indicated that all variables were significantly associated with tumour-positive margins (*Table 2*). Therefore, binary logistic regression including all variables was performed. ORs exceeding 1.5 for tumour-positive margins were observed for four characteristics: cT category, histological type, multifocal disease, and patients after neoadjuvant systemic therapy without pCR. Highest ORs were observed for cT3 tumours (OR 2.96; 95% c.i. 2.46 to 3.57), invasive lobular carcinoma (OR 2.84; 95% c.i. 2.62 to 3.08), and mixed-type tumours (OR 2.32; 95% c.i. 2.03 to 2.65). Furthermore, cT2 tumours (OR 1.56; 95% c.i. 1.46 to 1.66), multifocal disease (OR 1.94; 95% c.i. 1.79 to 2.09), and neoadjuvant systemic chemotherapy (OR 1.68; 95% c.i. 1.52 to 1.87) or hormonal therapy (OR 1.88; 95% c.i. 1.62 to 2.17) were found to be associated with significantly higher risks of tumour-positive margins. To a lesser extent, with ORs between 1.0 and 1.5, differentiation grade 2/3, the presence of DCIS in addition to invasive carcinoma, centrally located, and palpable tumours were found to increase the risk of tumour-positive margins. Alongside high-risk variables, significantly lower ORs were found for HR-negative tumours (HR−/HER2+ OR 0.57 (95% c.i. 0.46 to 0.72); HR−/HER2− OR 0.49 (95% c.i. 0.43 to 0.55)) and a preoperative MRI scan (OR 0.91; 95% c.i. 0.85 to 0.96). Subsequently, multinomial logistic regression was performed to evaluate relative risks (RRs) for focally and extensively positive margins separately. Variables with high ORs in binary regression also had higher RRs in multinomial regression for focally and extensively positive margins. Among these variables, cT3 tumours stood out, with an RR > 4.0 (RR 4.41; 95% c.i. 3.46 to 5.63; *Table 2*).

**Table 2:** Univariate and multivariable analysis: preoperative clinicopathological features correlated with margin status in women treated with breast-conserving surgery for non-metastatic breast cancer in the Netherlands from 2009 to 2022

	Univariate analysis		Multivariable analysis*		Multivariable analysis†			
	Total positive		Total positive		Focally positive‡		Extensively positive§	
	Odds ratio	*P*	Odds ratio	*P*	Relative risk	*P*	Relative risk	*P*
**Age (years)**								
< 40	1		1		1		1	
40–49	1.12 (0.99, 1.27)	0.073	1.11 (0.92, 1.33)	0.266	1.11 (0.88, 1.38)	0.376	1.11 (0.84, 1.48)	0.469
50–75	0.84 (0.75, 0.94)	0.003	1.00 (0.84, 1.19)	0.983	1.06 (0.86, 1.31)	0.592	0.89 (0.68, 1.17)	0.399
> 75	0.99 (0.87, 1.12)	0.852	1.11 (0.92, 1.35)	0.288	1.18 (0.93, 1.49)	0.179	1.00 (0.73, 1.36)	0.989
**Histological type**								
No special type	1		1		1		1	
Invasive lobular carcinoma	2.99 (2.85, 3.15)	< 0.001	2.84 (2.62, 3.08)	< 0.001	2.54 (2.31, 2.79)	< 0.001	3.52 (3.11, 4.00)	< 0.001
Mixed type	2.60 (2.37, 2.85)	< 0.001	2.32 (2.03, 2.65)	< 0.001	2.02 (1.72, 2.38)	< 0.001	3.00 (2.46, 3.65)	< 0.001
Other	0.88 (0.79, 0.98)	0.019	1.08 (0.94, 1.24)	0.280	0.99 (0.83, 1.17)	0.871	1.30 (1.03, 1.63)	0.024
**Multifocal disease**								
No	1		1		1		1	
Yes	2.30 (2.18, 2.43)	< 0.001	1.94 (1.79, 2.09)	< 0.001	1.90 (1.73, 2.09)	< 0.001	1.99 (1.76, 2.25)	< 0.001
**cT category**								
T1	1		1		1		1	
T2	1.92 (1.84, 2.00)	< 0.001	1.56 (1.46, 1.66)	< 0.001	1.49 (1.38, 1.61)	< 0.001	1.71 (1.54, 1.90)	< 0.001
T3	5.77 (5.16, 6.44)	< 0.001	2.96 (2.46, 3.57)	< 0.001	2.16 (1.70, 2.75)	< 0.001	4.41 (3.46, 5.63)	< 0.001
**Differentiation grade**								
1	1		1		1		1	
2	1.74 (1.66, 1.83)	< 0.001	1.28 (1.20, 1.37)	< 0.001	1.25 (1.16, 1.36)	< 0.001	1.35 (1.21, 1.52)	< 0.001
3	1.30 (1.23, 1.39)	< 0.001	1.24 (1.13, 1.35)	< 0.001	1.17 (1.05, 1.30)	0.003	1.40 (1.21, 1.62)	< 0.001
**DCIS component**								
No	1		1		1		1	
Yes	0.89 (0.86, 0.93)	< 0.001	1.19 (1.12, 1.26)	< 0.001	1.17 (1.09, 1.25)	< 0.001	1.23 (1.12, 1.36)	< 0.001
**Tumour palpable**								
No	1		1		1		1	
Yes	1.65 (1.57, 1.74)	< 0.001	1.30 (1.22, 1.38)	< 0.001	1.30 (1.21, 1.40)	< 0.001	1.31 (1.18, 1.45)	< 0.001
**Localization**								
Lateral	1		1		1		1	
Medial	0.98 (0.93, 1.03)	0.329	1.02 (0.96, 1.10)	0.510	1.03 (0.95, 1.12)	0.421	1.00 (0.89, 1.12)	0.975
Central	1.30 (1.20, 1.40)	< 0.001	1.23 (1.11, 1.37)	< 0.001	1.19 (1.05, 1.35)	0.006	1.32 (1.11, 1.57)	0.001
Other	1.18 (1.13, 1.24)	< 0.001	1.09 (1.02, 1.16)	0.009	1.03 (0.95, 1.12)	0.424	1.21 (1.09, 1.34)	< 0.001
**HR/HER2 status**								
HR+/HER2−	1		1		1		1	
HR+/HER2+	0.91 (0.84, 0.98)	0.016	0.90 (0.81, 1.02)	0.091	0.91 (0.79, 1.04)	0.161	0.90 (0.74, 1.10)	0.308
HR−/HER2+	0.62 (0.53, 0.73)	< 0.001	0.57 (0.46, 0.72)	< 0.001	0.63 (0.49, 0.81)	< 0.001	0.46 (0.31, 0.70)	0.001
HR−/HER2−	0.54 (0.49, 0.59)	< 0.001	0.49 (0.43, 0.55)	< 0.001	0.49 (0.42, 0.57)	< 0.001	0.49 (0.40, 0.60)	< 0.001
**Preoperative MRI scan**								
No	1		1		1		1	
Yes	1.50 (1.44, 1.56)	< 0.001	0.91 (0.85, 0.96)	0.002	0.90 (0.83, 0.97)	0.005	0.93 (0.84, 1.03)	0.159
**Neoadjuvant systemic therapy**								
None	1		1		1		1	
Chemotherapy	2.31 (2.18, 2.45)	< 0.001	1.68 (1.52, 1.87)	< 0.001	1.57 (1.38, 1.78)	< 0.001	1.89 (1.62, 2.21)	< 0.001
Hormonal therapy	2.97 (2.71, 3.24)	< 0.001	1.88 (1.62, 2.17)	< 0.001	1.70 (1.42, 2.03)	< 0.001	2.23 (1.80, 2.76)	< 0.001
Targeted therapy	1.27 (1.13, 1.43)	< 0.001	1.03 (0.84, 2.26)	0.778	1.07 (0.84, 1.36)	0.587	0.97 (0.69, 1.36)	0.859

Values in parentheses are 95% confidence intervals. *Binary logistic regression analysis. †Multinomial logistic regression analysis. ‡Defined as ≤ 4-mm tumour margin involvement. §Defined as > 4-mm tumour margin involvement or multiple focally positive foci. cT, clinical tumour; DCIS, ductal carcinoma *in situ*; HR, hormone receptor; HER2, human epidermal growth factor receptor 2; MRI, magnetic resonance imaging.

The multivariable models were translated into web-based prediction tools, which are available at https://www.evidencio.com/models/show/11737 for total positive margins (focally positive and extensively positive combined) and at https://www.evidencio.com/models/show/11739 for extensively positive margins only.

Information regarding invasive lobular carcinoma (invasive lobular carcinoma only/mixed-type), multifocal disease, cT2/cT3 tumour, and neoadjuvant chemotherapy or hormonal therapy without pCR is routinely known before surgery. Among patients without any of these risk factors, the adjusted probability of a positive surgical margin was 6.8% (95% c.i. 6.5 to 7.0%). The adjusted probability of tumour-positive margins increased with the number of risk factors: 12.4% (95% c.i. 11.9 to 12.8%) with one, 20.2% (95% c.i. 19.0 to 21.3%) with two, 33.5% (95% c.i. 30.8 to 36.1%) with three, and 49.1% (95% c.i. 39.9 to 58.2%) in patients with all four risk factors (*Table 3*).

**Table 3:** Adjusted probability with 95% confidence intervals for positive surgical margins, stratified by margin status and the number of risk factors identifiable before surgery (risk count)

Risk count*	Adjusted probability (%)†		
	Total positive	Focally positive‡	Extensively positive§
0	6.8 (6.5, 7.0)	4.7 (4.5, 4.9)	2.0 (1.9, 2.2)
1	12.4 (11.9, 12.8)	8.4 (8.0, 8.8)	4.0 (3.7, 4.3)
2	20.2 (19.0, 21.3)	12.7 (11.8, 13.7)	7.3 (6.6, 8.0)
3	33.5 (30.8, 36.1)	16.8 (14.7, 18.9)	16.0 (13.8, 18.2)
4	49.1 (39.9, 58.2)	28.0 (19.6, 36.4)	19.7 (12.4, 27.0)

Values in parentheses are 95% confidence intervals. *The risk count is defined as the unweighted sum of four binary variables: lobular carcinoma (including mixed type), multifocal disease, clinical tumour (cT) category ≥ 2, and the receipt of neoadjuvant chemotherapy or hormonal therapy without a pathological complete response. The risk count ranges from 0 (none) to 4 (all factors present). †Adjusted for age, differentiation grade, localization, ductal carcinoma *in situ* component, palpability, receptor status and preoperative magnetic resonance imaging. ‡Defined as ≤ 4-mm tumour margin involvement. §Defined as > 4-mm tumour margin involvement or multiple focally positive foci.

**Fig. 3:**
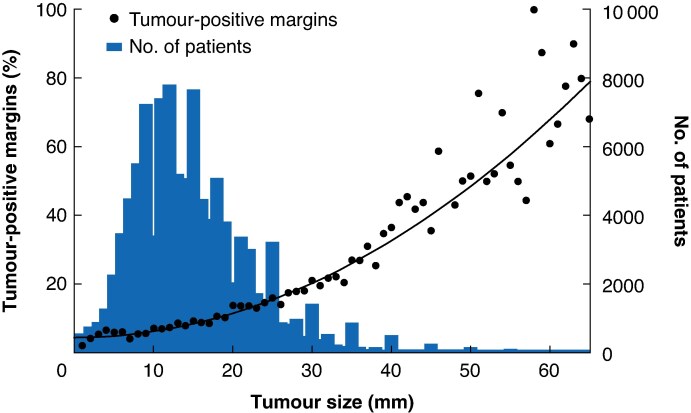
Rate of tumour-positive margins according to tumour size Tumour size was determined by pathology. The rate of tumour-positive margins relative to tumour size is shown by individual symbols; least-squares regression analysis was used to find the line of best-fit. Histograms show the number of patients per tumour size.

Following multivariable analysis, cT category appears to be the most important factor related to positive margins. Therefore, an in-depth analysis was performed of tumour size (in millimetres), as determined by pathology. This analysis showed that larger tumours had higher tumour-positive margin rates (*Fig. 3*).

In addition, the concordance between cT and pT categories and the relationship to positive margin rates was assessed. As indicated in *Table 4*, cT and pT categories were concordant in 80.9% of patients. In the remaining patients, 12 419 tumours (11.5%) were overstaged (cT>pT) and 8278 tumours (7.6%) were understaged (cT<pT). After adjustment for tumour, patient, and treatment characteristics, logistic regression showed an OR of 3.69 (95% c.i. 3.41 to 3.99) for positive margins in understaged tumours. Adjusted probability analysis showed that understaging tumours is consistently associated with the highest probabilities for positive margins, whereas overstaging tumours was associated with lower probabilities compared with concordant staging. Interestingly, preoperative MRI scans were performed in 60% of overstaged patients, compared with approximately 25% of patients with concordant or understaged tumours.

**Table 4:** Concordance (cT=pT), underestimation (cT<pT), and overestimation (cT>pT) of tumour size, stratified by margin status, with adjusted probabilities for positive surgical margins

	Total no. of patients (%)	All positive surgical margins		Focally positive surgical margins*		Extensively positive surgical margins†	
		No. of patients (%)	Adjusted probability‡ (%)	No. of patients (%)	Adjusted probability‡ (%)	No. of patients (%)	Adjusted probability‡ (%)
All patients	108 271 (100.0%)	11 733 (10.8%)		7548 (7.0%)		4185 (3.9%)	
**cT1**							
Concordant	72 850 (67.3%)	5286 (7.3%)	7.4	3736 (5.1%)	5.4	1550 (2.1%)	2.0
Understaged	7879 (7.3%)	1799 (22.8%)	21.9	1024 (13.0%)	11.9	775 (9.8%)	9.4
Overstaged	0 (0%)	0 (0%)	NA	0 (0%)	NA	0 (0%)	NA
**cT2**							
Concordant	14 451 (13.3%)	2535 (17.5%)	15.9	1558 (10.8%)	9.6	977 (6.8%)	5.8
Understaged	399 (0.4%)	282 (70.7%)	39.6	97 (24.3%)	20.2	185 (46.4%)	23.3
Overstaged	11 376 (10.5%)	1328 (11.7%)	9.5	906 (8.0%)	7.1	422 (3.7%)	2.7
**cT3**							
Concordant	273 (0.3%)	169 (61.9%)	29.4	41 (15.0%)	12.3	128 (46.9%)	16.0
Understaged	0 (0%)	0 (0%)	NA	0 (0%)	NA	0 (0%)	NA
Overstaged	1043 (1.0%)	334 (32.0%)	18.8	186 (17.8%)	9.2	148 (14.2%)	7.9

Values are *n* (%) unless otherwise stated. *Defined as ≤ 4-mm tumour margin involvement. †Defined as > 4-mm tumour margin involvement or multiple focally positive foci. ‡Adjusted for histology, multifocality, neoadjuvant treatment, age, differentiation grade, localization, ductal carcinoma *in situ* component, palpability, receptor status and preoperative magnetic resonance imaging. cT, clinical tumour; pT, pathological tumour; NA, not available.

### Margin status in high-risk subgroups over time

**Fig. 4:**
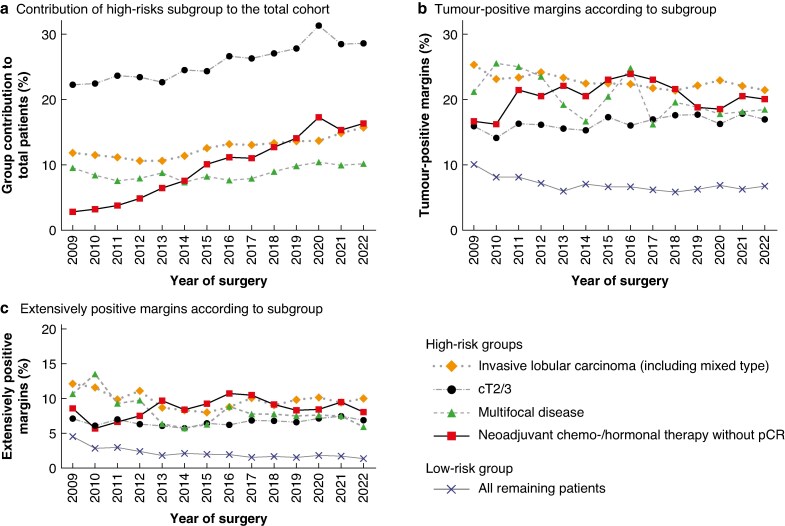
Comparisons of high-risk and low-risk subgroups among the cohort of women treated with breast-conserving surgery for non-metastatic breast cancer in the Netherlands from 2009 to 2022 **a** Percentage contribution of each high-risk subgroup to the total cohort per year. **b** Tumour-positive (combined focally and extensively positive margins) rates and **c** extensively positive margin rates in the high-risk and low-risk subgroups according to year of surgery. cT, clinical tumour; pCR, pathological complete response.

Patients with invasive lobular carcinoma (invasive lobular carcinoma only/mixed-type), multifocal disease, cT2/3 tumours, and pretreated with neoadjuvant chemotherapy or hormonal systemic therapy without pCR were found to be at the highest risk of tumour-positive margins. There were 44 772 patients with at least one of these characteristics, representing 40.9% of all patients included in the study; of these patients, 37 458 (83.7%) had negative margins and 7314 (16.3%) had positive margins (10.0% focally positive; 6.4% extensively positive). The percentage contribution to the total cohort for all high-risk characteristics, particularly cT2/3 category and pretreatment with neoadjuvant systemic therapy, increased over time (*Fig. 4a*). Tumour-positive margin rates (*Fig. 4b,c*), both total and extensively positive margins, are clearly higher in the high-risk subgroups than in the low-risk group (patients without any high-risk characteristics). Although a decline in tumour-positive margin rates was seen in the low-risk group up to 2013, no specific temporal pattern was seen in the high-risk subgroups.

## Discussion

This study analysed margin status after BCS for non-metastatic breast cancer in the Netherlands. From 2009 to 2022, 10.8% of patients had tumour-positive margins (6.9% focally positive; 3.9% extensively positive). Although the number of BCSs performed increased, rates of tumour-positive margins declined until 2013, and then stabilized at around 10.6%. This decline was primarily driven by a decrease in extensively positive margins. Moreover, this study identified four main factors resulting in higher risks of tumour-positive margins: invasive lobular carcinoma, multifocal disease, cT2/3 tumours, and neoadjuvant chemotherapy or hormonal systemic therapy without pCR (with ORs between 1.56 and 2.96). The probability of positive margins increased with the number of risk factors present, ranging from 6.8% in patients without risk factors to 49.1% in those with all four risk factors. Notably, patients with one or more of the high-risk characteristics accounted for 40.9% of the total study population. Furthermore, preoperative understaging (cT<pT) was associated with a higher risk of tumour-positive margins (OR 3.69). The multivariable analyses, including clinicopathological variables, have been translated into two web-based prediction tools (total positive margins: https://www.evidencio.com/models/show/11737; extensively positive margins: https://www.evidencio.com/models/show/11739). However, the tools require external validation and are not yet suitable for use in clinical decision-making.

The tumour-positive margin rate of 10.8% found in this study is comparable to that reported by a large meta-analysis^7^ of 68 studies comprising 112 140 patients, in which the tumour-positive rate was 9.4% (95% c.i. 6.8 to 12.8). In the literature, positive margin rates are diverse, ranging from 10% to 70%^5,6,17^, with most studies reporting rates between 20% and 40%. In contrast to these studies, patients with pCR were excluded, because the aim of the present study was to assess the potential impact of intraoperative margin assessment innovations^12,13,18^. If the 5747 patients with pCR had been included in the present study, assuming their margins were negative, the mean positive margin rate would likely be somewhat lower (∼10.3%). Although the tumour-positive margin rate of 10.8% in the present study is acceptable according to Dutch standards based on earlier quality indicators^19^, the authors believe that upcoming intraoperative margin assessment techniques, such as fluorescence imaging^12,13,18^, could considerably lower tumour-positive margin rates. Identified high-risk subgroups, which are comparable to those found in earlier studies in the Netherlands^20,21^ and the USA^17^, comprising 1185 and 3975 patients, respectively, could particularly benefit from intraoperative margin assessment techniques to reduce the need for boost radiotherapy and reoperations.

This study used extensive nationwide standardized data over a 14-year period, which is a strength. Nevertheless, several limitations should be acknowledged. The data were collected retrospectively from medical records and relied on hospital documentation and correct interpretation by trained IKNL staff, which may be subject to errors or missing data. In the analysis workflow, patients with missing data were excluded rather than using predictive imputation, which may reduce information bias but does not fully eliminate it. Residual confounding is possible, because factors such as breast/cup size, breast density, oncoplastic surgery or surgeons’ and pathologists’ experience, and guideline adherence were not recorded and may have influenced tumour-positive margin rates. Because these variables were not recorded, confounding could not be determined. In addition, centre-level variation in imaging availability, surgical practice, and pathology assessment could have influenced tumour-positive margin rates. Despite these limitations, the large number of patients and the detailed clinical variables support the overall reliability of the conclusions.

This large cohort study indicates that, in the Netherlands, positive margin rates decreased from 2009 to 2013, primarily driven by a decrease in extensively positive margins. In subsequent years, the overall rate stabilized at approximately 10.6%. Although these rates are comparable to those reported internationally, direct comparison is limited by differences in margin definitions and treatment strategies over time. Although many countries adopted the Society of Surgical Oncology’s ‘no ink on tumour’ guideline in 2014^22^, this principle has been used in the Netherlands since 2002, with an additional distinction between focally positive and extensively positive margins to guide adjuvant treatment strategies^9^. The differences in the clinical management of positive margins may influence long-term outcome after BCS, but the fundamental surgical goal of achieving a complete resection is shared internationally. Therefore, especially for data from 2014 onwards, the current findings, identifying high-risk subgroups for tumour-positive margins, are comparable and applicable in a broader, international context.

The decrease in extensively positive margins in the Netherlands until 2013 may be attributed to several factors. First, adherence to the guideline implemented in 2002 was gradual, resulting in a delayed reduction of reported extensively positive margin rates. Second, in addition to other outcomes, those of BCS at the hospital level have been made available by the National Breast Cancer Consultation Netherlands (NABON) Breast Cancer Audit (NBCA) since 2011, providing surgeons with margin involvement feedback and enabling comparisons between institutes. Finally, the tumour-positive margin rate became a publicly available quality indicator in 2011^23^. It is hypothesized that both the NBCA and broader awareness contributed most to the decrease in rates of extensive tumour-positive margins, by prompting surgeons to be more cautious in patient selection during the early years.

In the present study, the rate of tumour-positive margins has stabilized since 2014. This may partially reflect a clinical, patient-tailored approach where surgeons, in certain cases, operate with smaller margins and accept possible focally positive margins to preserve cosmetic outcomes. This is supported by the literature indicating that local recurrence and survival rates are comparable to those of patients after complete excision, provided that radiation, including boost, is administered for focally positive margins^10,24,25^. Furthermore, increasing the use of preoperative imaging techniques, such as MRI, contrast-enhanced mammography, and digital breast tomosynthesis, may have improved decision-making on BCS *versus* mastectomy. However, because data on mastectomies were not available, their impact remains speculative. Importantly, the plateau in tumour-positive margin rates over time may suggest that this represents the maximum achievable BCS result with current standards of care. This is especially relevant considering that more patients have undergone BCS over time, including those with complex indications. The approximately linear increase in the proportion of patients receiving neoadjuvant systemic therapy may be associated with a higher risk of positive margins. Stable rates despite these challenges may reflect ongoing improvements in surgical technique and patient selection. One of the surgical techniques contributing to this stabilization is the oncoplastic surgical technique, which enables more extensive tissue removal and improved cosmetic outcomes, thereby potentially reducing the incidence of positive margins. However, because data regarding surgical technique were not collected in this study, this hypothesis could not be examined.

High tumour-positive margin rates in specific subgroups and the fact that 40.9% of patients have at least one high-risk factor highlight the importance of acknowledging these. First, invasive lobular carcinomas are known for their characteristic non-cohesive growth pattern^26^, leading to cell clusters radiating outward from the main tumour, often with little host reaction or disturbance of background architecture. Therefore, tumour extensions are often difficult to delineate or not visible on preoperative imaging^27^. Higher positive margin rates for larger tumours may be explained by the larger total surface area, resulting in higher chances of surgical error. Multifocal disease, with numerous tumour foci that are neither visible nor palpable during surgery, makes complete tumour removal challenging. Finally, patients who underwent neoadjuvant systemic therapy without pCR had higher rates of positive margins, particularly those with ER-positive tumours. Potential explanations for this include patient selection and subtype-specific shrinkage patterns^28–30^. These shrinkage patterns result from heterogeneous responses to neoadjuvant systemic treatment, leading to multiple small tumour foci of residual disease that are difficult to detect with preoperative imaging. The predominance of ER-positive tumours in the positive margin group (92%) may be attributed, in part, to the exclusion of patients with a pCR, with higher rates of complete response in triple-negative and HER2-positive tumours, but also reflects the unique shrinkage and imaging characteristics associated with ER-positive disease. Because the present study also revealed a higher rate of tumour-positive margins in case of tumours understaged (cT<pT) before surgery, the importance of accurate preoperative staging and the selective use of intraoperative support is underscored. Preoperative MRI was performed more frequently in patients with overstaged tumours, a group consistently associated with lower tumour-positive margin rates. Nevertheless, MRI was also performed in 25% of patients with understaged tumours and did not reliably identify the true preoperative T category in these patients. Therefore, MRI may not be the ideal strategy to prevent positive surgical margins, although it may still provide benefit in select patients at risk of understaging. In addition to higher-risk subgroups, an unexpected finding of this study was that palpable tumours carry a significantly increased risk of positive margins, suggesting that intraoperative reliance on tumour palpability may be insufficient to delineate tumour boundaries. Emerging technologies, such as fluorescence imaging with tumour-specific fluorescent dyes^13,18^, optical coherence tomography^31^, and micro positron emission tomography/computed tomography scanning^32^, or other techniques such as imprint cytology/rapid on-site evaluation^33^, may improve the intraoperative determination of margins and facilitate the complete excision of tumours.

The results of the present study may guide shared-decision making and surgical planning. During 2009–2022, the mean tumour-positive margin rate in the Netherlands was 10.8%. Clinically relevant extensively positive margins, necessitating reoperation, were present in 3.9% of patients. In 6.9% of patients a focally positive margin was present, requiring adjuvant radiotherapy with a boost. Although this radiotherapy boost is deemed safe in terms of local recurrence and overall survival, associated patient burden (decreased cosmetic outcome, fibrosis with consequent pain and reduction in quality of life) should not be underestimated^8,10,34^. This study underscores that patients with cT2/3 tumours, invasive lobular carcinomas, multifocal disease, and patients who undergo neoadjuvant chemotherapy or hormonal therapy and do not reach a pCR remain at higher risk of tumour-positive margins. These subgroups comprised 40.9% of all patients. Therefore, there is still room for improvement.

## Data Availability

The data that support the findings of this study are available from the corresponding author upon reasonable request.

## Collaborators

Members of the NBCA consortium are: Martinus A. Beek (Isala, Zwolle, the Netherlands), Henriette A. Schuttevaer (Martini Hospital, Groningen, the Netherlands), Thomas Schok (VieCuri Medical Centre for North-Limburg, Venlo, the Netherlands), Hinne A. Rakhorst, Liesbeth Jansen (University Medical Centre Groningen, Groningen, the Netherlands), Marjan van Hezewijk (Institution for Radiation Oncology, Arnhem, the Netherlands), Carolien H. M. van Deurzen (Erasmus MC Cancer Institute, Rotterdam, the Netherlands), José H. Volders (Diakonessenhuis, Utrecht, the Netherlands), Ilse Jannink (Haga Hospital, The Hague, the Netherlands), Margrethe S. Schlooz-Vries (Radboud University Medical Centre, Nijmegen, the Netherlands), Ernst J. P. Schoenmaeckers (Meander MC, Amersfoort, the Netherlands), Marije C. Gordinou de Gouberville (Wilhelmina Hospital, Assen, the Netherlands), Alwine A. Hellingman (Tergooi MC, Hilversum, the Netherlands), Emily L. Postma (St. Antonius Hospital, Utrecht/Nieuwegein, the Netherlands), Ester J. M. Siemerink (Hospital Group Twente, Hengelo, the Netherlands), Floris P. R. Verbeek (Groene Hart Hospital, Gouda, the Netherlands), Marian B. E. Menke-Pluijmers (Albert Schweitzer Hospital, Dordrecht, the Netherlands), Joost Nonner (Ikazia Hospital, Rotterdam, the Netherlands), Vivianne C. G. Tjan-Heijnen (Maastricht University Medical Centre, Maastricht, the Netherlands), Yvonne L. J. Vissers (Zuyderland Medical Centre, Heerlen, the Netherlands), Linda de Munck (Netherlands Comprehensive Cancer Organisation (IKNL), Utrecht, the Netherlands), Djaëlla V. Hooiveld (Zaans Medical Centre, Zaandam, the Netherlands), Carlijn T. I. de Betue (Jeroen Bosch Hospital, ‘s-Hertogenbosch, the Netherlands), Dominique J. P. van Uden (Canisius Wilhelmina Hospital, Nijmegen, the Netherlands), Marianne R. F. Bosscher (Ommelander Hospital Groningen, Scheemda, the Netherlands), Marieke E. Straver (Haaglanden Medical Centre, The Hague, the Netherlands), Carla Meeuwis (Rijnstate Hospital, Arnhem, the Netherlands), Caroline M. E. Contant (Maasstad Hospital, Rotterdam, the Netherlands), Enja J. Bantema-Joppe (Radiotherapeutisch Instituut Friesland, Leeuwarden, the Netherlands), Milou H. Martens (Laurentius Hospital, Roermond, the Netherlands), Miriam L. Hoven-Gondrie (Hospital Gelderse Vallei, Ede, the Netherlands), Léonie H. M. Smit (Treant Zorggroep Hospital, Hoogeveen, the Netherlands), Marthe Brinckman (Frisius MC, Leeuwarden, the Netherlands), Gea A. Gooiker (Northwest Hospital Group, Alkmaar, the Netherlands).

## References

[1] Libson S, Koshenkov V, Rodgers S, Hurley J, Avisar E. Breast conservation after neoadjuvant therapy for tumors ≥ 5 cm: a prospective cohort study. Int J Surg Open 2015;1:10–13

[2] Mauriac L, Macgrogan G, Avril A, Durand M, Floquet A, Debled M et al Neoadjuvant chemotherapy for operable breast carcinoma larger than 3 cm: a unicentre randomized trial with a 124-month median follow-up. Ann Oncol 1999;10:47–52 10076721 10.1023/a:1008337009350

[3] De Lorenzi F, Loschi P, Bagnardi V, Rotmensz N, Hubner G, Mazzarol G et al Oncoplastic breast-conserving surgery for tumors larger than 2 centimeters: is it oncologically safe? A matched-cohort analysis. Ann Surg Oncol 2016;23:1852–1859 26842491 10.1245/s10434-016-5124-4

[4] Dillon MF, Mc Dermott EW, O'Doherty A, Quinn CM, Hill AD, O’Higgins N. Breast oncology factors affecting successful breast conservation for ductal carcinoma in situ. Ann Surg Oncol 2007;14:1618–1628 17443388 10.1245/s10434-006-9246-y

[5] Pleijhuis RG, Graafland M, De Vries J, Bart J, de Jong JS, van Dam GM et al Obtaining adequate surgical margins in breast-conserving therapy for patients with early-stage breast cancer: current modalities and future directions. Ann Surg Oncol 2009;16:2717–2730 19609829 10.1245/s10434-009-0609-z PMC2749177

[6] Jacobs L . Positive margins: the challenge continues for breast surgeons. Ann Surg Oncol 2008;15:1272 10.1245/s10434-007-9766-0 PMC2277448 18320287

[7] Bundred JR, Michael S, Stuart B, Cutress RI, Beckmann K, Holleczek B et al Margin status and survival outcomes after breast cancer conservation surgery: prospectively registered systematic review and meta-analysis. BMJ 2022;378:e070346 36130770 10.1136/bmj-2022-070346 PMC9490551

[8] King MT, Link EK, Whelan TJ, Olivotto IA, Kunkler I, Westenberg AH et al Quality of life after breast-conserving therapy and adjuvant radiotherapy for non-low-risk ductal carcinoma in situ (BIG 3-07/TROG 07.01): 2-year results of a randomised, controlled, phase 3 trial. Lancet Oncol 2020;21:685–698 32203696 10.1016/S1470-2045(20)30085-1

[9] National Breast Cancer Consultation Netherlands (NABON) . Breast Cancer Dutch Guideline. Version 2.0. NABON, 2012. https://www.nabon.nl/wp-content/uploads/2022/10/Dutch-Breast-Cancer-Guideline-2012.pdf (accessed 20 December 2024)

[10] Vos EL, Siesling S, Baaijens MHA, Verhoef C, Jager A, Voogd AC et al Omitting re-excision for focally positive margins after breast-conserving surgery does not impair disease-free and overall survival. Breast Cancer Res Treat 2017;164:157–167 28389735 10.1007/s10549-017-4232-6 PMC5487695

[11] Volders JH, Haloua MH, Krekel NMA, Meijer S, van den Tol PM. Current status of ultrasound-guided surgery in the treatment of breast cancer. World J Clin Oncol 2016;7:44 26862490 10.5306/wjco.v7.i1.44 PMC4734937

[12] Koller M, Qiu SQ, Linssen MD, Jansen L, Kelder W, de Vries J et al Implementation and benchmarking of a novel analytical framework to clinically evaluate tumor-specific fluorescent tracers. Nat Commun 2018;9:1–11 30228269 10.1038/s41467-018-05727-y PMC6143516

[13] Voskuil FJ, Vonk J, van der Vegt B, Kruijff S, Ntziachristos V, van der Zaag PJ et al Intraoperative imaging in pathology-assisted surgery. Nature Biomedical Eng 2021;6:503–514 10.1038/s41551-021-00808-8 34750537

[14] Veluponnar D, de Boer LL, Dashtbozorg B, Jong L-JS, Geldof F, Guimaraes MDS et al Margin assessment during breast conserving surgery using diffuse reflectance spectroscopy. J Biomed Opt 2024;29:045006 38665316 10.1117/1.JBO.29.4.045006 PMC11045169

[15] Casparie M, Tiebosch ATMG, Burger G, Blauwgeers H, van de Pol A, van Krieken JHJM et al Pathology databanking and biobanking in the Netherlands, a central role for PALGA, the nationwide histopathology and cytopathology data network and archive. Cellular oncol 2007;29:19–24 10.1155/2007/971816 PMC4618410 17429138

[16] Rakha EA, El-Sayed ME, Lee AHS, Elston CW, Grainge MJ, Hodi Z et al Prognostic significance of Nottingham histologic grade in invasive breast carcinoma. J Clin Oncol 2008;26:3153–3158 18490649 10.1200/JCO.2007.15.5986

[17] Chagpar AB, Martin RCG, Hagendoorn LJ, Chao C, McMasters KM. Lumpectomy margins are affected by tumor size and histologic subtype but not by biopsy technique. Am J Surg 2004;188:399–402 15474434 10.1016/j.amjsurg.2004.06.020

[18] Schouw HM, Huisman LA, Janssen YF, Slart RHJA, Borra RJH, Willemsen ATM et al Targeted optical fluorescence imaging: a meta-narrative review and future perspectives. Eur J Nucl Med Mol Imaging 2021;48:4272–4292 34633509 10.1007/s00259-021-05504-y PMC8566445

[19] Gooiker GA, Veerbeek L, van der Geest LGM, Stijnen T, Dekker JWT, Nortier JWR. De prestatie-indicator ‘irradicaliteit na borstsparende operatie’. Geen zuiver zicht op goede zorg. Ned Tijdschr Geneeskd 2010;154:A1142 20482902

[20] Pleijhuis RG, Kwast ABG, Jansen L, de Vries J, Lanting R, Bart J et al A validated web-based nomogram for predicting positive surgical margins following breast-conserving surgery as a preoperative tool for clinical decision-making. Breast 2013;22:773–779 23462681 10.1016/j.breast.2013.01.010

[21] Pleijhuis RG, Kwast ABG, Jansen L, de Vries J, Wiggers T, Siesling S et al Validity of the BreastConservation! nomogram evaluated. Breast 2015;24:540–542 26346587 10.1016/j.breast.2015.07.001

[22] Moran MS, Schnitt SJ, Giuliano AE, Harris JR, Khan SA, Horton J et al Society of Surgical Oncology–American Society for Radiation Oncology consensus guideline on margins for breast-conserving surgery with whole-breast irradiation in stages I and II invasive breast cancer. J Clin Oncol 2014;32:1507–1515 24516019 10.1200/JCO.2013.53.3935

[23] van Bommel ACM, Spronk PER, Vrancken Peeters MJTFD, Jager A, Lobbes M, Maduro JH et al Clinical auditing as an instrument for quality improvement in breast cancer care in the Netherlands: the national NABON breast cancer audit. J Surg Oncol 2017;115:243–249 27885679 10.1002/jso.24516

[24] Houssami N, Macaskill P, Marinovich ML, Morrow M. The association of surgical margins and local recurrence in women with early-stage invasive breast cancer treated with breast-conserving therapy: a meta-analysis. Ann Surg Oncol 2014;21:717–730 24473640 10.1245/s10434-014-3480-5 PMC5705035

[25] Vos EL, Jager A, Verhoef C, Voogd AC, Koppert LB. Overall survival in patients with a re-excision following breast conserving surgery compared to those without in a large population-based cohort. Eur J Cancer 2015;51:282–291 25549530 10.1016/j.ejca.2014.12.003

[26] Reed MEMC, Kutasovic JR, Lakhani SR, Simpson PT. Invasive lobular carcinoma of the breast: morphology, biomarkers and ‘omics. Breast Cancer Res 2015;17:12 25849106 10.1186/s13058-015-0519-x PMC4310190

[27] Johnson K, Sarma D, Hwang ES. Lobular breast cancer series: imaging. Breast Cancer Res 2015;17:1–8 26163296 10.1186/s13058-015-0605-0 PMC4499185

[28] Tarantino P, Hortobagyi G, Tolaney SM, Mittendorf EA. Heterogeneity of residual disease after neoadjuvant systemic therapy in breast cancer: a review. JAMA Oncol 2024;10:1584 10.1001/jamaoncol.2024.3679 39264638

[29] Wang M, Du S, Gao S, Zhao R, Liu S, Jiang W et al MRI-based tumor shrinkage patterns after early neoadjuvant therapy in breast cancer: correlation with molecular subtypes and pathological response after therapy. Breast Cancer Res 2024;26:1–15 38347619 10.1186/s13058-024-01781-1 PMC10863121

[30] Fukada I, Araki K, Kobayashi K, Shibayama T, Takahashi S, Gomi N et al Pattern of tumor shrinkage during neoadjuvant chemotherapy is associated with prognosis in low-grade luminal early breast cancer. Radiology 2018;286:49–57 28737968 10.1148/radiol.2017161548

[31] Erickson-Bhatt SJ, Nolan RM, Shemonski ND, Adie SG, Putney J, Darga D et al Real-time imaging of the resection bed using a handheld probe to reduce incidence of microscopic positive margins in cancer surgery. Cancer Res 2015;75:3706–3712 26374464 10.1158/0008-5472.CAN-15-0464 PMC4749141

[32] Lambert B, Vergucht V, Dekeyser S, De Craene A, Ameye F, Van Den Bossche B et al Feasibility study on the implementation of a mobile high-resolution PET/CT scanner for surgical specimens: exploring clinical applications and practical considerations. Eur J Nucl Med Mol Imaging 2025;52:2979–2994 39976699 10.1007/s00259-025-07143-z

[33] Satturwar S, Rekhtman N, Lin O, Pantanowitz L. An update on touch preparations of small biopsies. J Am Soc Cytopathol 2020;9:322–331 32417160 10.1016/j.jasc.2020.04.004 PMC8375623

[34] Bartelink H, Maingon P, Poortmans P, Weltens C, Fourquet A, Jager J et al Whole-breast irradiation with or without a boost for patients treated with breast-conserving surgery for early breast cancer: 20-year follow-up of a randomised phase 3 trial. Lancet Oncol 2015;16:47–56 25500422 10.1016/S1470-2045(14)71156-8

